# The Research Process of PSK Biosynthesis, Signaling Transduction, and Potential Applications in *Brassica napus*

**DOI:** 10.3390/plants12173075

**Published:** 2023-08-28

**Authors:** Xuwen Shen, Nils Stührwohldt, Chen Lin

**Affiliations:** 1 Key Laboratory of Plant Functional Genomics of the Ministry of Education, Yangzhou University, Yangzhou 225009, China; 15862056944@163.com; 2 Department of Plant Physiology and Biochemistry, Institute of Biology, University of Hohenheim, 70593 Stuttgart, Germany; n.stuehrwohldt@mvz-labor-lb.de

**Keywords:** phytosulfokine, growth regulator, physiological functions, signal transduction, rapeseed

## Abstract

Phytosulfokine (PSK) is a disulfated pentapeptide that acts as a growth regulator to control plant growth and development as well as adaptability to biotic and abiotic stress. In the last three decades, PSK has drawn increasing attention due to its various functions. Preproproteins that have been tyrosine sulfonylated and then cleaved by specific enzymes contribute to mature PSK. To transfer a signal from the apoplast to the inner cells, the PSK peptide must bind to the PSK receptors (PSKR1 and PSKR2) at the cell surface. The precise mechanism of PSK signal transduction is still unknown, given that PSKR combines receptor and kinase activity with a capacity to bind calmodulin (CaM). The binding of PSK and PSKR stimulates an abundance of cGMP downstream from PSKR, further activating a cation-translocating unit composed of cyclic nucleotide-gated channel 17 (CNGC17), H^+^-ATPases AHA1 and AHA2, and BRI-associated receptor kinase 1 (BAK1). Recently, it has been revealed that posttranslational ubiquitination is closely related to the control of PSK and PSKR binding. To date, the majority of studies related to PSK have used *Arabidopsis*. Given that rapeseed and *Arabidopsis* share a close genetic relationship, the relevant knowledge obtained from *Arabidopsis* can be further applied to rapeseed.

## 1. Introduction

Phytosulfokine (PSK) is a disulfated pentapeptide that has been identified as a growth factor in plants. PSK was initially isolated from the mesophyll cells of *Asparagus officinalis* in a culture medium [1]. It was hypothesized that PSK was made up of two components, PSK-α and PSK-β, that differed from each other due to a tiny structural variation. More recently, experimental evidence has demonstrated that PSK-β is the degradational byproduct of PSK-α [2]. PSK–γ and PSK-ε, both of which belong to the PSK family, have recently been identified in soybean (*Glycine max*) and *Medicago truncatula*, respectively [3,4]. Similarly to most small, post-translationally modified peptides, mature and active PSK that is derived from a precursor preproprotein has to be modified and cleaved. PSK is a precursor protein that must be sulfonated at both tyrosines by tyrosylprotein subfotransferase (TPST), localized in the Golgi apparatus, and further cleaved by subtilisin-like serine proteases (SBTs) in the cytoplasmic matrix. AtSBT1.1 and AtSBT3.8 have been demonstrated to participate in the proteolytic cleavage of AtPSK4 and AtPSK1 propeptides, respectively [5,6,7]. It is still unknown whether other SBT family members or even unidentified proteases are also involved in the modification of PSK preproproteins. After processing, mature PSK binds to the PSK receptor proteins (PSKRs) localized at the plasma membrane. PSKRs have a canonical guanylate cyclase (GC) core and act upstream of cGMP and PSK signaling; therefore, they must undergo sophisticated signal transduction within cells. The regulation of signal transduction is also mediated by the response modules of cyclic nucleotide-gated channel 17 (CNGC17), H^+^-ATPase AHA1 and AHA2, and BRI-associated receptor kinase 1 (BAK1) on the plasma membrane [8].

Several putative functions of PSK have been reported, including the regulation of plant growth and development, callus formation, sexual reproduction, plant response to biotic and abiotic stress. Research on PSK biosynthesis and signal transduction has advanced dramatically over the last three decades. PSK, emerging as an important small signaling molecule, is receiving increasing global attention. The majority of studies on PSK have so far focused on *Arabidopsis* and rice (*Oryza sativa*). Despite being incredibly potent and crucial for plants, the physiological functions of PSK are currently not employed in crops. The PSK precursor genes belong to a rather conservative gene family. *Brassica napus* (*B. napus*) and *Arabidopsis* are genetically closely related. *B. napus* contains 24 PSK homologous genes based on the sequence alignment of *AtPSK1*-*AtPSK7* in *Arabidopsis*, and these genes are expressed in different plant tissues. This raises the prospect of enhancing the endogenous PSK content in *B. napus* to either stimulate yield production or enhance plant tolerance to biotic and abiotic stress. 

This review summarizes research on PSK biosynthesis, signal transduction, and the physiological roles of PSK, along with a discussion about the future of PSK-related research. Additionally, potential applications are suggested for genetic transformation, stress tolerance, and cultivation of elite germplasm resources in rapeseed, which might be beneficial for molecular breeding and genetic modification in agriculture. 

## 2. Synthesis and Signal Transduction of PSK Peptide

### 2.1. Biosynthesis of the PSK Peptide

The majority of peptides, which are derived from precursor proteins, go through a series of posttranslational modifications, including tyrosine sulfation, proline hydroxylation, glycosylation, and processing by proteases, before becoming active molecules that ultimately cause physiological responses [9,10]. Seven precursor genes (*AtPSK1-AtPSK7*) encoding PSK have been identified in *Arabidopsis*. *AtPSK1* shows the highest expression level in the root and seed. The expression of the *AtPSK2* and *AtPSK3* genes is detectable not only in suspension-cultured cells but also throughout the entire plant, with the highest expression in the root [11]. *AtPSK4* is primarily expressed in the root, hypocotyl, seed, leaf, and flower, with the highest expression in the senescent leaf and mature seed. *AtPSK5* is mainly expressed in the flower, and the expression of *AtPSK6* is comparatively low in most plant tissues. *AtPSK7* is specifically expressed in the silique and seed (according to the online database https://www.arabidopsis.org/ (accessed on 20 June 2023)). These PSK precursor genes appear to be differentially expressed in diverse plant tissues and at different plant developmental phases. 

**Figure 1:**
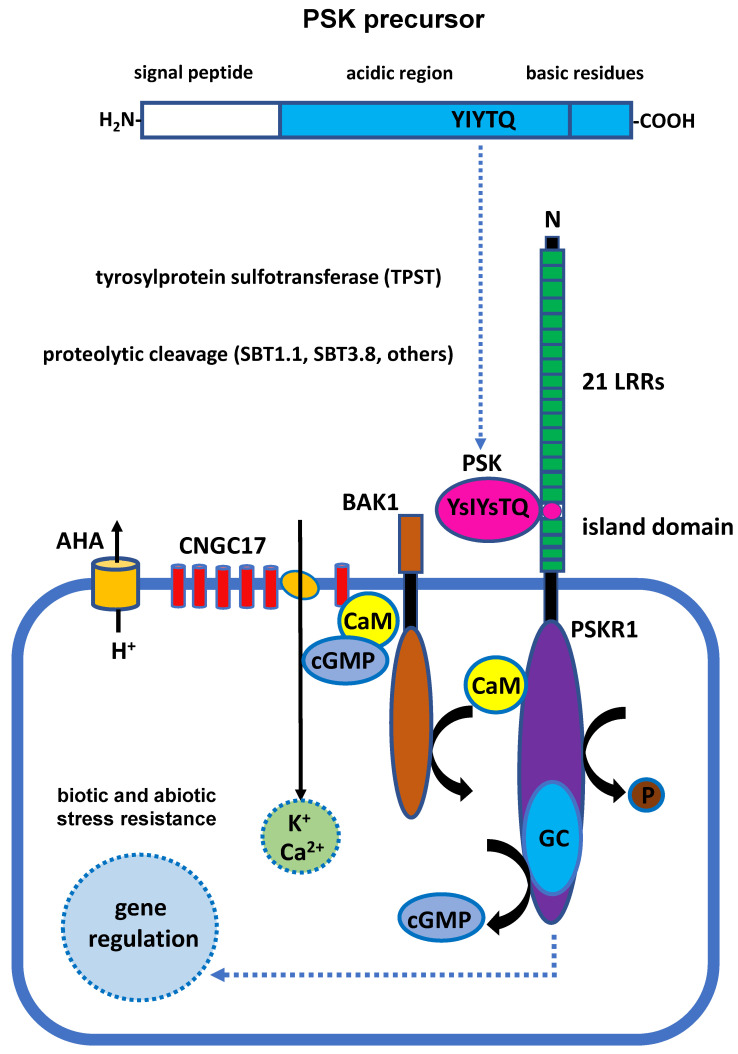
Schematic model of PSK biosynthesis and signaling cascade uncovered in *Arabidopsis*. The N-terminus of the PSK precursor protein contains a hydrophobic amino acid residue, and the characteristic sequence YIYTQ/H is located at the C-terminus of the precursor protein. YIYTQ/H is surrounded by two pairs of alkaline amino acid residues that might be important for precursor processing. The precursor protein is first sulfonated by TPST localized on the Golgi apparatus membrane to modify two tyrosines of sequence YIYTQ/H. The sulfonated preproprotein is further cleaved by SBTs in the cytoplasmic matrix. After maturation of PSK, it is secreted into the extracellular apoplast and recognized by the extracellular LRR domains of PSKR. PSKR also contains cyclic guanylate kinase domain and a calmodulin (CaM) binding site in the intracellular kinase domain of PSKR; the production of cGMP is therefore stimulated after the binding of PSK and PSKR. Finally, the downstream signal controls cell growth and development through gene expressional regulation, while the control of kinase activity occurs through the binding of calcium ions and calmodulin (CaM). In addition, potassium ions are also indispensable for PSK signal transduction. cGMP acts as the second messenger downstream of PSKR1 to activate CNGC17. Instead of a direct interaction between PSKR1 and CNGC17, they rather form a complex module with the H^+^-ATPases AHA1 and AHA2 in the plasma membrane, as well as BRI-associated receptor kinase 1 (BAK1). This schematic view was modified by Sauter and Ladwig [8,14].

PSK precursor proteins contain 80–120 amino acids, which are members of a small protein family [12]. The N-terminus of the precursor protein consists of hydrophobic leader residues for protein secretion, while the PSK signature motif YIYTQ/H is located at the C-terminus of the precursor protein and surrounded by two pairs of basic residues [2,12]. Tyrosylprotein sulfotransferase (TPST), which is localized on the Golgi membrane, is required for the sulfonylation of both tyrosines on the unique PSK motif YIYTQ/H as part of the processing of PSK preproprotein [5]. The sulfonated precursor protein leaves the Golgi apparatus and is further cleaved by subtilisin-like serine proteases in the cytoplasmic matrix (Figure 1). In *Arabidopsis*, it has been demonstrated that AtSBT1.1 and AtSBT3.8 specifically cleave and modify two different PSK preproproteins, AtPSK4 and AtPSK1. The processing of the AtPSK4 propeptide relies on AtSBT1.1 localized in the apoplast [6]. Recent research has indicated that extracellular AtSBT3.8 cleaves AtPSK1 propeptide in an aspartate-specific manner [13] because the cleavage by AtSBT3.8 relies on aspartic acid residue at the C-terminus [7]. It is still necessary to identify the proteases involved in the modification of the N-terminus of the PSK precursor in *Arabidopsis*. The SBT family contains 56 members, and it is likely that other SBTs (which require further investigation in the future) are also involved in the cleavage of PSK precursor proteins. 

### 2.2. Characteristics of PSK Receptors

PSK is an autocrine growth regulator produced in the secretory pathway and further released into the extracellular apoplast [1]. The typical phytohormones and regulatory factors in plants are perceived by receptors and conveyed through signaling transduction pathways to elicit a response within plant cells. Earlier studies have described the specific binding sites with PSK in the plasma membrane cells of carrots (*Daucus carota*), corn (*Zea mays*), asparagus (*Asparagus officinalis*), and tomatoes (*Solanum lycopersicum*) using radioisotope labeling [15,16,17], indicating that this type of receptor is present in almost all higher plants. Soon after, via affinity chromatography, it is shown that carrot cells contain a phytosulfokine receptor (PSKR) with a molecular weight of 120 KD that can specifically bind to PSK [18,19]. The PSKR is localized in the plasma membrane [1]. Extracellular leucine-rich repeats (LRRs), a transmembrane domain, and a cytoplasmic kinase domain are three components that contribute to an integrated and functional PSKR [18,19]. PSKRs are categorized as members of the RLK (receptor-like kinase) protein family that contains a chemical structure with receptor-like kinases [20]. In addition, PSKRs and brassinosteroid receptor BRI1 (brassinosteroid receptor BRASINOSTEROID INSENSITIVE 1) belong to the LRR X RLK subfamily and play a key role in the regulation of plant growth and development [20]. The extracellular LRRs are composed of 21 consecutive LRR domains, and each LRR domain contains 24 amino acid residues. A highly independent island domain with 36 amino acid residues is found between the 17th and 18th LRR domains [18] (Figure 1). The island domain composed of 36 amino acid residues is highly conserved on AtPSKR1 and the brassinosteroid receptor BRI1 [18,21], suggesting that this domain plays a key role in the interplay between PSK and PSKR. In *Arabidopsis*, two PSKR homologous genes have been discovered [22,23]. 

### 2.3. PSKR-Dependent Signal Transduction 

Two PSK receptors are present in *Arabidopsis*: AtPSKR1 and AtPSKR2 [22,23]. Through protein kinase activity assay in vitro and bioinformatic study of these two receptors, it has been revealed that AtPSKR1 has a cyclic guanylate kinase domain and a calmodulin (CaM) binding site [24,25]. Therefore, the interaction between PSK and PSKR promotes the synthesis of cGMP, which then transmits downstream signal transduction and, ultimately, regulates cell growth and development. In contrast, the activity of kinase is mediated by the binding of calcium ions and CaM. In addition, potassium ion is indispensable for the transmission of PSK signals. It is shown that PSK fails to induce protoplast swelling in the absence of exogenous potassium ions [26]. PSK-induced protoplast expansion decreases by approximately 60% at culture medium with 1 mM K^+^ as compared with the expansion with 10 mM K^+^ in a dose-dependent manner [26]. 

Cyclic nucleotide-gated channels (CNGCs) are one of the targets regulated by cGMP in plant cells [27], and CNGC17 specifically promotes protoplast expansion. Moreover, CNGC17 is also co-expressed with PSKR1 to control seedling growth [8]. Therefore, it is likely that PSK activates the cation channel CNGC17 and exerts physiological effects in conjunction with a second messenger, cGMP, which is located downstream of PSKR1. Instead of directly interacting with CNGC17, PSKR1 promotes a physiological response with CNGC17 by forming a protein complex on the plasma membrane with the H^+^-ATPases AHA1 and AHA2, as well as BAK1 [8] (Figure 1). The relevant studies above indicate that PSK is recognized by PSKR after the precursor protein is processed, matured, and secreted. Afterward, the transmitted PSK signaling further controls plant cell growth and development, as well as plants’ response to environmental stresses through hormone signals, second messengers, ion concentration changes, etc. However, the underlying molecular mechanism is not yet known in detail and requires much more experimental investigation. 

Recently, it was discovered that the ubiquitin/proteasome degradation pathway is the mechanism by which the PSKR1 protein activity is mediated. In the absence of PSK, PSKR1 is moderately ubiquitinated as a result of interactions between PSKR1 and plant U-box E3 ligases, PUB12 and PUB13, that regulate PSKR1 abundance in response to pathogen infection. In the presence of PSK, PSKR1 is unable to interact with PUB12 and PUB13, thereby activating PSK-mediated intercellular defense signals [28]. This study highlights how a PSK-PSKR pattern is dynamically regulated by post-translational regulation, laying the foundation for a deeper comprehension of the intricate PSK signaling transduction system.

## 3. Physiological Functions of PSK

### 3.1. PSK Controls the Growth and Development of Plant Cells 

In plants, the function of PSK on cell proliferation has been widely reported (Table 1). In the suspension culture of mesophyll cells of *Asparagus officinalis*, PSK enhances mitotic events for cell proliferation at the nanomolar level [1]. Relevant studies in rice [15] and carrot cells [29] further support a comparable function. In addition, PSK stimulates the growth of adventitious roots on the hypocotyl of cucumber (*Cucumis sativus*)[30] and accelerates the development of somatic cell embryos in carrot and *Cryptomeria japonica* [5,31], indicating a physiological role of PSK in promoting cell differentiation and somatic embryogenesis. Recent research also confirmed that PSK contributes to early somatic embryogenesis in *Cunninghamia lanceolate*. PSK not only boosts the number of somatic cell embryos but also hastens their growth and maturation [32]. Moreover, this PSK-stimulating effect is genotype-independent in *Cunninghamia lanceolata* and can be employed to generate a somatic embryogenesis system for various genotypes. PSK also controls root elongation and fiber development. Root cells at the quiescent center (QC), which undergo delayed cell division, are responsible for maintaining the meristem of the root tip [33,34,35]. Continuous root growth mostly occurs at the root meristem and elongation zone. PSK regulates root elongation via enhanced cell expansion with brassinosteroids synergistically in *Arabidopsis* [36,37,38]. Compared to wild-type plants, *AtPSKR* double mutant plants have shorter roots and hypocotyls, while overexpressing *AtPSKR1* in plants significantly enhances the root length [26,38]. TPST plays an important role in the sulfonylation of PSK precursor proteins; *tpst* mutants in *Arabidopsis* exhibit a dwarf phenotype accompanied by severe shoot and root development [39]. Additionally, mutant plants that are deficient in *AtSBT3.8* exhibit a substantial decrease in root development, whereas PSK treatment recovers the root growth [7]. The exogenous application of synthesized PSK has been examined in the growth system of cotton (*Gossypium species*) ovules at various concentrations in vitro, and it has been verified that PSK exhibits a positive impact on the elongation of cotton fiber, while GA_3_ is required to reach that end [40]. The wheat (*Triticum aestivum*) gene *TaPSK5* is newly identified as a target of microRNA164 (miR164). *TaPSK5*, which encodes a PSK precursor protein, enhances root elongation and boosts crop yield [41]. PSK-γ isolated in soybean is a functional analog of PSK and increases both seed size and weight, and is, therefore, thought to be favorable to higher soybean yield [42]. 

Pollen density determines pollen germination and pollen tube elongation in most plants, which is referred to as the “pollen population effect”. The frequency of pollen germination is closely related to the pollen density and is supported by studies performed with mature pollen grains in tobacco (*Nicotiana tabacum*) suspended in culture medium. The low pollen germination efficiency can be reversed using a conditioned medium from the pollen culture, and the presence of PSK is further confirmed in the conditioned medium using an enzyme-linked immunosorbent assay, supporting the involvement of PSK as a key regulatory factor in the pollen population effect [43]. It has been demonstrated that PSK is able to promote early embryogenesis of microspores in *B. napus* [44]. In addition, it is revealed that PSK signaling is essential for pollen tube growth in *Arabidopsis*, redirecting them from the transmitting tract to the embryo sac properly [45]. So far, the understanding of the structural characteristics and functions of PSK remains limited in Rosaceae species. It is shown that the overexpression of the *PbrPSK2* gene in *Pyrus bretschneideri* promotes pollen tube elongation [46]. These findings imply that PSK is a multifunctional regulator for plant growth and development, and the modulation of PSK-related gene expression has considerable potential for crop improvement.

**Table 1:** The physiological functions of PSK in plants.

Major Function	Function in Detail	Species	Reference
PSK controls the growth and development of plant cells	cell proliferation	*Asparagus officinalis,* *Oryza sativa,* *Daucus carota*	[1]; [15]; [29]
	induced cell differentiation and somatic cell embryogenesis	*Cucumis sativus,* *Daucus carota,* *Cryptomeria japonica,* *Cunninghamia lanceolata*	[30]; [5]; [31]; [32]
	regulation of root elongation and fiber development	*Arabidopsis,* *Gossypium species,* *Triticum aestivum*	[36]; [40]; [41]
	increased crop yield	*Triticum aestivum,* *Glycine max*	[42]; [41]
	regulatory factor in pollen population effects	*Nicotiana tabacum*	[43]
	guide pollen into the embryo sac and enable them to elongate normally	*Arabidopsis* *Pyrus bretschneideri*	[45]; [46]
PSK promotes callus formation and efficiency of tissue culture in vitro	induced cell regeneration	*Brassica oleracea*	[47]
	enhanced the transformation efficiency of candidate genes	*Daucus carota*	[48]
PSK regulates plant adaptation in response to environmental stresses	enhanced heat resistance	*Arabidopsis*	[49]
	enhanced drought resistance	*Oryza sativa,* *Arabidopsis* *Solanum lycopersicum*	[50]; [7]; [51]
	delayed senescence	*Fragaria ×ananassa,* *Rosa hybrida,* *Brassica oleracea* var. *Italica*	[52]; [53]; [54]
	preserved the quality and delayed cold damage	*Musa paradisiaca,* *Prunus persica*	[55]; [56]
	pathogen resistance	*Solanum lycopersicum,* *Arabidopsis,* *Oryza sativa*	[28,57]; [58,59]; [60]
	balance plant immune signaling	*Arabidopsis*	[61]

### 3.2. PSK Promotes Callus Formation and Efficiency of Tissue Culture In Vitro

It has been extensively documented that PSK is involved in the regulation of callus development (Table 1). Transgenic callus tissues containing the constitutively expressed *AtPSK2* or *AtPSK3* chimeric genes double in size when compared with the mock callus [11], suggesting that PSK plays a role in assisting callus formation. The protoplasts of sugar beet (*Beta vulgaris* L.) cannot differentiate into callus tissue in vitro due to their recalcitrant status; however, PSK is able to reverse this scenario, and it enhances cell division in most sugar beet cultivars [62]. Surprisingly, 0.1 µM PSK is sufficient to trigger the mitotic event and noticeably induce the cell regeneration of protoplasts in *Brassica oleracea* in vitro [47]. Additionally, PSK vastly enhances the transformation efficiency in carrots, and the majority of the transformed callus successfully differentiate into cotyledons and roots before maturing into full-grown plants [48]. A substantial guarantee for the tissue regeneration of the transformation system can be provided by PSK, which improves callus formation and transformation efficiency on genetic manipulation in plants.

### 3.3. PSK Regulates Plant Adaptation in Response to Environmental Stresses

Plenty of research has shown how PSK improves plant tolerance to biotic and abiotic stress (Table 1). PSK improves plants’ ability to withstand the heat in *Arabidopsis*, based on the phenotypic analysis of plants subjected to high night-time temperature conditions with or without PSK treatment [49]. It is further revealed that PSK delays the senescence of strawberries (*Fragaria × ananassa*) stored at 4 °C [52], the petal senescence of cut rose flowers (*Rosa hybrida* cv. Angelina), [53] and the yellowing of broccoli (*Brassica oleracea* var. Italica) florets [54] due to improved antioxidant capacity. Furthermore, the quality of bananas (*Musa paradisiaca*) and peaches (*Prunus persica*) is also retained, and cold damage is postponed owing to the exogenous application of PSK [55,56]. These research results suggest that PSK plays a role in plant assimilation of environmental challenges, which is desirable for the development of the agricultural industry. The growth and yield of crops have been threatened in recent decades by the escalating severity of drought events in addition to heat and cold stress. To adapt to these environmental stresses, plants have had to develop various adaptive traits or mechanisms. Premature flower drop in tomato largely reduces the yield production under drought conditions. Phytaspase (Phyt) is a subtilisin-like protease of the phytaspase subtype. It has been revealed that *SlPSK1*, *SlPSK6,* and *SlPhyt2* are co-expressed at the abscission zone of tomato flower organs. The knockout of *SlPhyt2* results in delayed abscission of flowers under drought stress, indicating that PSK signaling is involved in drought-induced flower drop [51]. Recent studies have reported that the expression of PSK precursor genes and three subtilisin-like serine proteases, SBT1.4, SBT3.7, and SBT3.8, are highly induced by osmotic stress [7]. In the *sbt3.8* loss-of-function mutant, the processing of AtPSK1 propeptide is impaired, and mature PSK could not be produced properly. The roots of the *sbt3.8* mutant are significantly shorter than those of the wildtype seedlings combined with a decreased fresh weight of the roots and number of lateral roots under osmotic stress conditions, finally resulting in decreased plant tolerance to osmotic stress. This phenomenon is largely alleviated with PSK treatment. Contrarily, plants overexpressing *AtPSK1* or *AtSBT3.8* show an enhanced tolerance to osmotic stress [7]. The expressional analysis of *OsPSKR* genes using RNA-seq and real-time quantitative PCR (RT-qPCR) shows that multiple PSKR genes are differently elevated when induced by drought and salinity, demonstrating that *OsPSKR* genes are involved in rice adaptation to biotic and abiotic stress [50]. The resultant evidence indicates that PSK plays a significant role and develops a special mechanism to help plants deal with abiotic stresses.

Growing evidence suggests that PSK also participates in the plant immune response to various biotic stresses (Table 1). PSK is a danger-associated molecular pattern (DAMP), which is primarily recognized by PSKR1 [28,58]. To enhance tomato resistance to *B. cinerea*, PSKR1 elevates the Ca^2+^ concentration in the cytosol and activates the auxin-mediated signaling pathway [58]. The expression of *PSK2* and *PSKR1* is highly induced after the treatment of the fungal elicitor E-Fol or the fungal pathogens *Alternaria brassicicola* and *Sclerotinia sclerotiorum* on the leaves of *Arabidopsis*. The defense system of the *pskr1* mutant plant against fungus is largely impaired [59]. Further investigation reveals that overexpression of the PSK precursor gene *AtPSK2* or *AtPSK4* in *Arabidopsis* significantly enhances resistance to *Alternaria brassicicola* [57], indicating the role of PSK in plant immune response. The severe fungal disease *Xanthomonas oryzae* pv. *oryzicola* (*Xoc*), which results in bacterial leaf streak (BLS), brings the global rice supply toward danger of collapse. Overexpressing *OsPSKR1* in rice increases plant resistance to *Xoc* [60]. The expression of *PSK3*, *PSK5,* and *PSKR1* are wound-induced in hypocotyls and leaves [59]; these findings indicate that PSK regulates plant adaptation to biotic stress. Although the immune system acts as a shield to protect plants, a hyperactive response frequently has a detrimental and irreversible effect on plant growth [63,64]. In addition, the exogenous application of PSK attenuates pattern-triggered immunity (PTI) in *Arabidopsis*, therefore reducing excessively robust immunity [61]. Thus, it is plausible that PSK is essential for maintaining the proper balance between plant development and immune system responses.

## 4. PSK-Related Genetic Correlation between Rapeseed and *Arabidopsis*

To date, the model plant *Arabidopsis* has remained the focus of most PSK research. Rapeseed and *Arabidopsis* have a close genetic link, and both are members of the Brassicaceae family. As a result, the knowledge gained from research on *Arabidopsis* can be further transferred to rapeseed. The development of bioinformatics has allowed us to obtain sequence information regarding PSK across different rapeseed genotypes. 

**Figure 2:**
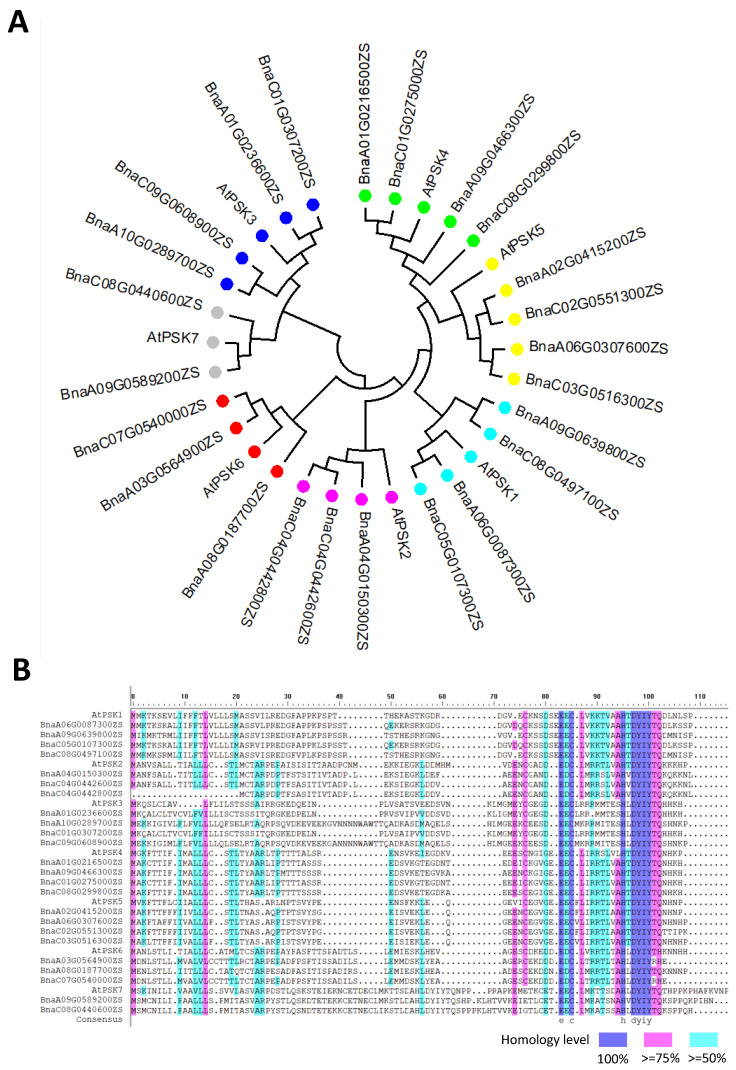
Alignment of PSK precursor proteins in *A. thaliana* and *B. napus.* (**A**) The Neighbor-Joining (NJ) phylogenetic tree regarding the PSK precursor proteins was constructed with MEGA7 software, based on amino acid alignment between *A. thaliana* and *B. napus*. Dots with the same color represent homologous proteins. This is an originally developed figure. (**B**) The amino acid alignment reveals the conserved domain in the PSK precursor proteins. The amino acid sequence was obtained from an online database (http://yanglab.hzau.edu.cn/BnTIR (accessed on 20 June 2023)). Sequence alignment was performed with DNAman8 software. This is an originally developed figure.

**Figure 3:**
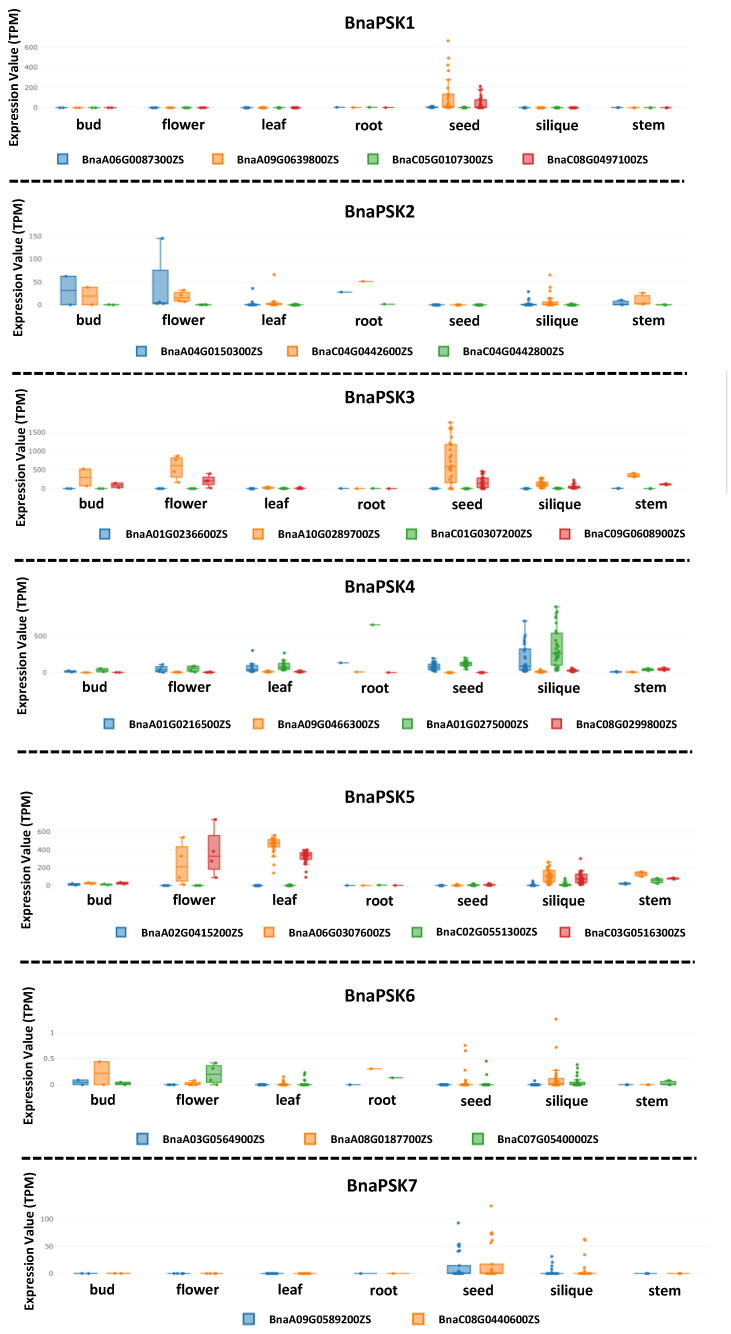
Expressional profiles of PSK precursor genes in different plant tissues of *B. napus*. The data of the expressional profiles were obtained from an online database with slight modifications (http://yanglab.hzau.edu.cn/BnTIR (accessed on 20 June 2023)).

Seven independent PSK precursor proteins with the PSK signature pattern YIYTQ/H have been identified in *Arabidopsis* and their corresponding genes are expressed in various plant tissues. PSK precursor genes are a relatively conservative gene family, with a high sequence similarity between *Arabidopsis* and rapeseed, based on the sequence alignment and phylogenetic tree analysis (Figure 2). The number of precursor genes in rapeseed that correspond to *AtPSK1-AtPSK7* in *Arabidopsis* is 4, 3, 4, 4, 4, 3, and 2 (Figure 2). According to the online database, these genes are expressed in various rapeseed tissues and organs. *BnaPSK1* is mostly expressed in seed, while *BnaPSK2* is expressed in flower buds, flowers, and roots. *BnaPSK3* is universally expressed in flower buds, flowers, and seeds. *BnaPSK4* is primarily expressed in pod, and *BnaPSK5* is mostly expressed in flower, leaf, and pod. In all tissues, the expression of *BnaPSK6* and *BnaPSK7* is comparatively low (Figure 3). The expressional patterns of the PSK precursor genes in rapeseed further confirm the idea that PSK signaling is present throughout rapeseed plants. This raises the prospect of enhancing the PSK content of particular tissues in rapeseed. Future work might explore the possibility of enhancing the promoter activity of various PSK precursor genes to boost the expression level of the corresponding genes.

## 5. Conclusions and Perspectives

### 5.1. Bottleneck and Gaps of PSK-Related Research

In *Arabidopsis*, substantial advances have been made in PSK biosynthesis, PSKR-dependent signal transduction, and PSK-related physiological functions. Nevertheless, it is necessary to address several concerns related to PSK in detail, as follows: (1) Of the 56 members of the SBT family of enzymes, only AtSBT1.1 and AtSBT3.8 were found to specifically cleave AtPSK4 and AtPSK1 propeptide. The modification or cleavage of PSK preproproteins most likely also involves additional SBTs or unidentified proteases [6,13]; (2) The precise regulatory mechanism of PSK signaling transduction is still unknown, despite the identification of several regulatory elements in the PSKR-dependent signal transduction; (3) Both phytohormones and PSK are essential for the regulation of plant development, while the interplay between PSK and these phytohormones is so far unclear; (4) The physiological applications of PSK to most crops are currently poorly studied due to the majority of PSK research being concentrated on *Arabidopsis*; (5) It has been recently revealed that PSKR1 interacts with the plant U-box E3 ligases PUB12 and PUB13, and the abundance and protein stability of PSKR are controlled by the ubiquitin/proteasome degradation pathway. It is, therefore, anticipated that the PSKR protein can be stabilized by repressing the activity of PUB12 or PUB13 for the improvement of plant disease resistance. However, it is unclear whether the knockout of PUB12 or PUB13 causes any other negative impacts on plants.

### 5.2. Putative Function of PSK on Callus Formation during Genetic Transformation in B. napus

Rapeseed is a crucial oil crop that stabilizes oil output on a global scale. Meanwhile, rapeseed plants at vegetative phases can also be served as edible vegetables. The largest and most popular variety of rapeseed grown worldwide is *B. napus*. Therefore, both domestically and globally, research into relevant economic and adaptive traits in *B. napus* is gaining popularity.

In rapeseed, *B. napus* exhibits a higher rate of genetic transformation than other *Brassica* species [65,66]. To date, the genetic transformation of *B. napus* still relies on agrobacterium-mediated hypocotyl transformation, and the transformation efficiency varies among different varieties. The regeneration rate of four commercial rapeseed varieties (Faisal canola, Punjab canola, Aari canola, and Nifa Gold) as well as the model variety (Westar) have been tested. Aari canola shows the highest transformation efficiency at 50.6%, followed by Westar with a transformation rate of 37.3%. The transformation efficiencies of other genotypes are still less than 33% [66]. In other academic research, the transformation efficiency of four different rapeseed varieties (GY284, WH3417, ZS9, and ZS11) after co-incubation with agrobacterium strain was found to be 3.7–5.6%, 1.1–3.5%, 0–0.87%, and 0, respectively [67], indicating that the genotypes in rapeseed continue to play a significant role in the efficiency of the agrobacterium-mediated transformation. It has been made clear that, in addition to genotypes, the transformation efficiency is also strongly correlated with the ratio of various hormones and the status of explants [68].

Previous research reported that a tiny amount of PSK considerably increases the transformation efficiency of various plants [47,48,62]. In order to improve the effectiveness of genetic transformation independent of genotypes in rapeseed, PSK can thus be evaluated in the genetic transformation. Experimental investigation is still needed to determine the optimal amount of PSK during tissue culture. In the meantime, it is anticipated that the overexpression of PSK precursor genes in rapeseed plants might bring about comparable outcomes.

### 5.3. Potential Use of PSK-Related Genes in Agricultural Breeding

PSK serves a variety of functions and is crucial for increasing crop yield and stress tolerance [7,50]. However, rapeseed has not exhibited these helpful attributes. The breakthrough in bioinformatics has made it possible for us to acquire sequence data about PSK across multiple rapeseed genotypes. It may be conceivable to investigate PSK-related quantitative trait loci (QTLs) or optimal haplotypes to improve plant growth and stress tolerance by combining phenotypic and molecular biology investigations in rapeseed. This work is closely associated with agricultural output in the future. 

PSKR1 is mainly ubiquitinated by PUB13 through Lys-748 and Lys-905 sites. The Lys-748 and Lys-905 sites of PSKR, which are important protein sites, can be manipulated as an alternative strategy to confer disease resistance. These modifications largely rely on base editing or prime editing technology. Future work will likely focus on the identification of PUB members that interact with PSKR1 in rapeseed. It is anticipated that the crucial ubiquitination sites can be modified to maintain the abundance and stability of PSKR1, which may help accelerate the cultivation of rapeseed resistant to bacterial and fungal infections. It is also reasonable to consider, at the molecular level, how to optimize the activity of the enzymes involved in posttranslational modifications, such as TPST and SBTs. Future studies need to meticulously evaluate the inhibitors that control the activity of these enzymes. The accurate genome editing of these suppressors aids in enhancing PSK activity or content, which may be crucial in the growth and development of rapeseed.

## Data Availability

Not applicable.
